# Stress Hyperglycemia Ratio Outperforms Glycemic Variability in Predicting Mortality Among Acute Myocardial Infarction Patients With Reduced Ejection Fraction: A Retrospective Cohort Study

**DOI:** 10.1111/1753-0407.70122

**Published:** 2025-08-13

**Authors:** Weiyan Lai, Xiu Ying Peng, Hui Peng, Yang Chen

**Affiliations:** ^1^ Department of Nephrology, The Third Affiliated Hospital Sun Yat‐sen University Guangzhou Guangdong China; ^2^ Department of Gastroenterology The Third Affiliated Hospital of Sun Yat‐sen University Guangzhou China; ^3^ Department of Cardiology, The Third Affiliated Hospital Sun Yat‐sen University Guangzhou Guangdong China

**Keywords:** acute myocardial infarction, glycemic variability, mortality, stress hyperglycemia ratio, triglyceride glucose index, ventricular tachycardia/ventricular fibrillation

## Abstract

**Aims:**

Stress hyperglycemia ratio (SHR) and glycemic variability (GV) are established markers of glucose metabolism dysregulation. This study compared their predictive values for mortality and arrhythmic events in patients with reduced ejection fraction following acute myocardial infarction (AMI).

**Materials and Methods:**

We analyzed 1933 AMI patients with reduced ejection fraction from the MIMIC‐IV database (v3.1, 2008–2022). The primary endpoint was in‐hospital mortality, with secondary endpoints including 1‐year all‐cause mortality, ventricular tachycardia/ventricular fibrillation (VT/VF), and cardiac arrest. Multivariate logistic regression models evaluated associations with in‐hospital outcomes, while Cox proportional hazards models assessed 1‐year mortality. Restricted cubic spline models examined non‐linear relationships between SHR and outcomes, with discriminative ability compared using area under the receiver operating characteristic curve (AUC) analysis.

**Results:**

Among patients (mean age 67.3 years), 401 (20.7%) died during hospitalization and 662 (34.2%) within one year. After adjustment, SHR showed the strongest association with in‐hospital mortality (odds ratio [OR]: 1.51, 95% confidence interval [CI]: 1.24–1.82) compared to GV (OR: 1.00, 95% CI: 0.99–1.01). For 1‐year mortality, SHR maintained superior performance (hazard ratio: 1.40, 95% CI: 1.19–1.65), with the highest tertile significantly associated with increased risk. ROC analysis confirmed SHR's superior predictive capacity for both mortality endpoints and VT/VF, while none of the indices significantly predicted cardiac arrest. SHR's predictive value was more pronounced in non‐diabetic patients.

**Conclusions:**

In post‐AMI patients with reduced ejection fraction, SHR demonstrated superior predictive value for mortality compared to GV, supporting its incorporation into risk stratification models for individualized glucose management.

## Graphical abstract

Stress hyperglycemia ratio outperforms glycemic variability and triglycerideȐglucose index in predicting mortality and arrhythmic events especially significant in non‐diabetic patients.

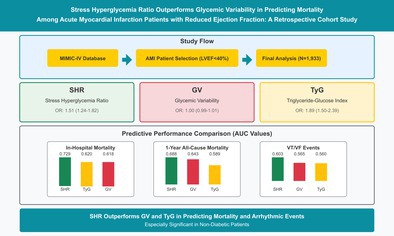

**Summary**

- SHR significantly outperforms GV and TyG index in predicting in‐hospital and 1‐year all‐cause mortality in AMI patients with reduced EF.
- SHR demonstrates superior predictive ability for VT/VF events with AUC of 0.603, better than GV and TyG.
- SHR's predictive value is more pronounced in non‐diabetic patients.
- Multivariate analysis shows each unit increase in SHR elevates in‐hospital mortality risk by 51% (OR:1.51, 95% CI:1.24–1.82).
- SHR should be incorporated into risk stratification models for AMI patients with reduced EF to guide individualized glycemic management.

## 1 Introduction

Patients with reduced ejection fraction following acute myocardial infarction (AMI) face significant mortality risk and adverse outcomes, particularly during ICU admission. In recent years, glucose metabolism disorders have been established as closely associated with the development and progression of cardiovascular diseases, especially in AMI patients, where glycemic abnormalities may exacerbate myocardial injury and increase the risk of arrhythmias and mortality [1, 2].

Stress hyperglycemia refers to the transient elevation of blood glucose levels in response to physiological or psychological stress, commonly observed in critically ill patients [3, 4, 5] The stress hyperglycemia ratio (SHR), which evaluates the relative degree of glucose elevation during acute illness compared to baseline glycemic status, better reflects the intensity of the body's stress response [6, 7] Glycemic variability (GV) reflects the degree of blood glucose fluctuation and has been recognized as an independent risk factor for diabetic complications [8, 9]. Studies have confirmed that both SHR and GV are closely associated with systemic inflammation, oxidative stress, and endothelial dysfunction, which contribute to adverse cardiovascular outcomes [10, 11, 12, 13]. Moreover, even transient exposure to stress hyperglycemia and acute high GV can have long‐term effects on cardiovascular events through inducing epigenetic modifications, a phenomenon known as the “metabolic memory phenomenon” [14]. Therefore, the assessment of stress hyperglycemia and GV is of great importance for glycemic management and improving prognosis.

However, comparative studies of these two glucose metabolism disorder indices in predicting mortality risk among patients with reduced ejection fraction after AMI remain scarce. This study aims to systematically compare the value of SHR and GV in predicting in‐hospital and one‐year all‐cause mortality in patients with reduced ejection fraction following AMI, to explore the associations between these indices and the risk of VT/VF and cardiac arrest, and to comparatively investigate the performance differences between these two glucose metabolism disorder indices and the triglyceride‐glucose index (TyG) in cardiovascular risk assessment.

## 2 Methods

### 2.1 Study Design and Population

This retrospective cohort study utilized data extracted from the Medical Information Mart for Intensive Care IV (MIMIC‐IV 3.1) database. MIMIC‐IV is a publicly available large‐scale clinical database containing detailed clinical information of critically ill patients treated at Beth Israel Deaconess Medical Center from 2008 to 2022. We included all adult patients diagnosed with AMI and left ventricular ejection fraction (LVEF) less than 40%. AMI diagnosis was based on International Classification of Diseases (ICD) codes (ICD‐9: 410.x or ICD‐10: I21.x) and confirmed through clinical records. LVEF values were obtained from discharge records.

### 2.2 Exclusion Criteria

Exclusion criteria included: (1) patients lacking glycated hemoglobin (HbA1c) data; (2) hospitalization duration less than 6 h; (3) patients with LVEF greater than 40%; and (4) patients with multiple hospitalizations. Patients with variables missing more than 30% of data were excluded from the analysis. For the remaining missing data, multiple imputation was employed to minimize bias.

### 2.3 Data Collection

Demographic characteristics, clinical features, laboratory test results, and treatment information were extracted from the MIMIC‐IV database. Demographic characteristics included age, sex, and race. Clinical features encompassed body mass index (BMI), systolic blood pressure (SBP), diastolic blood pressure (DBP), heart rate, medical history (diabetes, hypertension, stroke, heart failure [HF], atrial fibrillation [AF], chronic kidney disease [CKD], and chronic obstructive pulmonary disease [COPD]), and intensive care scoring systems. Laboratory test results included blood glucose, glycated hemoglobin, lipid profile, creatinine, lactate, blood gas analysis, blood cell counts, troponin T (TnT), N‐terminal pro‐brain natriuretic peptide (ProBNP), and LVEF. Treatment information comprised pharmacological interventions (angiotensin‐converting enzyme inhibitors/angiotensin receptor blockers [ACEI/ARB], β‐blockers, statins, aspirin, digoxin, and diuretics) and procedural interventions (percutaneous coronary intervention [PCI], coronary artery bypass grafting [CABG], intra‐aortic balloon pump [IABP], renal replacement therapy [CRRT], and invasive mechanical ventilation).

### 2.4 Metabolic Indices Definition

SHR was defined as the ratio of admission blood glucose level to estimated average glucose level, and the estimated average glucose level was calculated using the **formula: 28.7 × HbA1c−46.7 [15]. GV was defined as the coefficient of variation of blood glucose measurements during hospitalization [16]. The TyG index was calculated using the formula: Ln [fasting triglycerides (mg/dL) × fasting glucose (mg/dL)/2] [17].

### 2.5 Outcome Definition

The primary endpoints were in‐hospital and 1‐year all‐cause mortality. Mortality information was extracted from the electronic health record system. Secondary endpoints included the occurrence of cardiac arrest and VT/VF during hospitalization. These events were similarly extracted from electronic health records using ICD diagnostic codes and verified by two independent researchers. Cardiac arrest was defined as ICD‐9 code 427.5 or ICD‐10 code I46.x; VT/VF was defined as ICD‐9 codes 427.1, 427.4 or ICD‐10 codes I47.2, I49.0.

### 2.6 Statistical Analysis

In this study, the three metabolic indices (SHR, GV, and TyG) were analyzed as both continuous variables and in tertiles (*T*1–*T*3) to systematically compare their predictive performance for in‐hospital and 1‐year all‐cause mortality, as well as arrhythmic events in AMI patients with LVEF < 40%. Continuous variables were expressed as mean ± standard deviation or median (interquartile range) for non‐normally distributed data. Categorical variables were reported as frequencies and percentages. Between‐group differences were assessed using analysis of variance (ANOVA) for continuous variables and *χ* ^2^ tests for categorical variables. Variables with more than 30% missing data were excluded, and multiple imputation was used to handle remaining missing values.

Multivariate logistic regression models were employed to determine the relationship between the three indices and the risk of in‐hospital mortality, 1‐year all‐cause mortality, cardiac arrest, and VT/VF. We constructed three sequential models: an unadjusted model; Model 1, adjusted for age, sex, race, diabetes, stroke, HF, AF, CKD, and COPD; and Model 2, adjusted for the variables in Model 1 plus BMI, SBP, heart rate, hemoglobin, white blood cell count (WBC), platelet count (PLT), serum albumin, lactate, creatinine, partial pressure of oxygen (PaO_2_), triglycerides (TG), low‐density lipoprotein (LDL), high‐density lipoprotein (HDL), LVEF, sequential organ failure assessment score (SOFA), systemic inflammatory response syndrome index (SIRS), simplified acute physiology score II (SAPSII), acute physiology score III (APSIII), ACEI/ARB, statins, aspirin, digoxin, β‐blockers, diuretics, CRRT, invasive mechanical ventilation, PCI, CABG, and IABP.

Restricted cubic spline (RCS) analysis was used to examine the dose‐response association between SHR and outcomes of interest. Receiver operating characteristic (ROC) curve analysis was subsequently performed to compare the predictive ability, sensitivity, and specificity of these three indices for in‐hospital mortality, 1‐year all‐cause mortality, cardiac arrest, and VT/VF. A *p* value of < 0.05 was considered statistically significant. All statistical analyses were performed using Stata (version 16, STATA Corporation) and SPSS (version 21, IBM Corporation, USA) statistical software.

## 3 Results

### 3.1 Baseline Characteristics of Study Participants

The participant screening process for both cohorts is illustrated in Figure S1. From the MIMIC‐IV database, 1933 patients with AMI and LVEF < 40% who met the inclusion criteria were included in our analysis. The mean age was 67.26 ± 12.34 years, with 1277 (66.1%) being male. Table 1 presents the baseline characteristics according to quartiles of the SHR index during hospitalization. Patients in higher SHR quartiles were older and had a higher prevalence of stroke, AF, but a lower prevalence of diabetes and hypertension. They also demonstrated a higher heart rate, SBP, BMI, lactate, serum creatinine, partial pressure of carbon dioxide (PaCO_2_), WBC, PLT, TG, LDL, SOFA, SIRS, and APSIII score, but lower serum albumin, PaO_2_, and HDL levels.

**TABLE 1:** Participant characteristics of MIMIC‐IV database according to tertiles of SHR index at baseline.

Variables	Overall (*N* = 1933)	Quartile 1 (*N* = 645)	Quartile 2 (*N* = 644)	Quartile 3 (*N* = 644)	*p*
SHR	1.036 (0.905–1.221)	0.851 (0.749–0.905)	1.037 (0.986–1.078)	1.337 (1.221–1.575)	**< 0.001**
GV	22.83 (16.39–32.33)	23.11 (15.34–31.78)	20.02 (52.08–62.79)	25.84 (18.90–36.17)	**< 0.001**
TyG	9.17 ± 0.84	9.08 ± 0.91	9.10 ± 0.83	9.32 ± 0.76	**< 0.001**
*Demographics*					
Age (years)	67.26 ± 12.34	65.66 ± 12.70	67.21 ± 11.84	68.91 ± 12.27	**< 0.001**
Gender (male, *n* [%])	1277 (66.1%)	417 (64.8%)	451 (70.0%)	409 (63.5%)	0.031
Race (white, *n* [%])	1116 (57.7%)	358 (55.6%)	388 (60.2%)	370 (57.5%)	0.223
*Vital signs*					
SBP (mmHg)	117.52 ± 14.69	116.01 ± 14.60	117.41 ± 14.71	118.18 ± 14.33	**0.013**
DBP (mmHg)	56.89 (52.08–62.79)	56.32 (51.54–61.87)	56.73 (51.91–63.18)	56.30 (52.30–62.86)	0.057
Heart rate (bpm)	84.58 ± 11.40	82.73 ± 11.42	84.34 ± 11.09	84.80 ± 11.07	**< 0.001**
BMI (kg/m^2^)	30.33 ± 11.95	28.74 ± 12.72	30.10 ± 11.94	30.30 ± 9.23	**< 0.001**
*Comorbidities*					
Diabetes (*n* [%])	721 (37.3%)	327 (50.8%)	207 (32.1%)	187 (29.0%)	**< 0.001**
Stroke (*n* [%])	359 (18.6%)	95 (14.8%)	114 (17.7%)	150 (23.3%)	**< 0.001**
Hypertension (*n* [%])	724 (37.5%)	274 (42.5%)	252 (39.1%)	198 (30.7%)	**< 0.001**
HF (*n* [%])	808 (41.8%)	253 (39.3%)	257 (39.9%)	298 (46.3%)	**0.018**
AF (*n* [%])	792 (41.0%)	239 (37.1%)	252 (39.1%)	301 (46.7%)	**0.001**
CKD (*n* [%])	598 (30.9%)	192 (29.8%)	196 (30.4%)	210 (32.6%)	**0.514**
COPD (*n* [%])	239 (12.4%)	74 (11.5%)	69 (10.7%)	96 (14.9%)	0.051
*Laboratory tests and examination*					
Lactate (mmol/L)	1.89 (1.48–2.43)	1.76 (1.40–2.30)	1.83 (1.45–2.41)	1.93 (1.51–2.47)	**< 0.001**
Albumin (g/dL)	3.60 (3.20–4.00)	3.60 (3.20–4.00)	3.63 (3.23–4.00)	3.63 (3.20–4.00)	0.161
Creatinine (mg/dL)	1.32 ± 0.84	1.16 ± 0.64	1.28 ± 0.90	1.34 ± 0.82	**< 0.001**
PaO_2_ (mmHg)	159.61 ± 69.47	178.09 ± 74.80	164.11 ± 69.83	156.16 ± 66.93	**< 0.001**
PaCO_2_ (mmHg)	41.42 ± 7.10	40.69 ± 6.86	41.31 ± 7.32	41.54 ± 7.20	**0.004**
Hemoglobin (g/dL)	10.72 ± 1.47	10.79 ± 1.37	10.75 ± 1.47	10.80 ± 1.49	**0.016**
WBC (K/μL)	10.25 ± 5.13	9.48 ± 3.27	10.46 ± 7.38	10.36 ± 3.81	**< 0.001**
PLT (K/μL)	227.87 ± 83.63	218.95 ± 80.54	224.97 ± 84.49	234.36 ± 87.47	**0.011**
FPG (mg/dL)	131.77 ± 30.74	114.90 ± 18.35	125.03 ± 24.96	133.86 ± 27.29	**< 0.001**
TG (mg/dL)	118.20 (86.00–163.45)	71.00 (59.50–82.00)	104.00 (90.00–116.50)	141.29 (121.50–158.50)	**< 0.001**
LDL (mg/dL)	84.27 ± 32.03	73.33 ± 29.48	83.83 ± 31.72	86.13 ± 32.69	**< 0.001**
HDL (mg/dL)	47.71 ± 17.54	54.39 ± 17.53	49.61 ± 16.99	46.84 ± 16.57	**< 0.001**
HbA1c (%)	6.41 ± 1.71	7.49 ± 1.72	6.23 ± 1.17	5.51 ± 1.55	**< 0.001**
TnT (ng/mL)	0.59 (0.17–2.03)	0.74 (0.16–2.26)	0.61 (0.18–1.92)	0.49 (0.15–1.74)	**0.218**
ProBNP (pg/mL)	11538.38 (5181.00–20926.79)	10306.13 (4737.68–18628.16)	11288.70 (5181.00–19920.97)	13564.89 (5704.83–24300.00)	**< 0.001**
LVEF (%)	55.10 ± 13.95	54.13 ± 11.59	55.44 ± 11.44	55.74 ± 17.83	0.046
*Scoring systems*					
SOFA (*n* [%])	4.27 ± 2.64	4.08 ± 2.56	4.14 ± 2.50	4.35 ± 2.71	**0.023**
SIRS (*n* [%])	2.62 ± 0.77	2.50 ± 0.80	2.60 ± 0.77	2.69 ± 0.75	**< 0.001**
SAPSII (*n* [%])	40.89 ± 13.09	40.19 ± 12.88	41.23 ± 12.60	41.07 ± 13.36	0.482
APSIII (*n* [%])	47.44 ± 21.33	45.64 ± 20.61	45.27 ± 19.77	47.68 ± 21.90	**< 0.001**
*Prescription*					
ACEI/ARB (*n* [%])	336 (17.4%)	125 (19.4%)	115 (17.9%)	96 (14.9%)	0.098
Statin (*n* [%])	1648 (85.3%)	578 (89.8%)	573 (89.0%)	497 (77.2%)	**< 0.001**
Aspirin (*n* [%])	1697 (87.8%)	592 (91.9%)	591 (91.8%)	514 (79.8%)	**< 0.001**
Digoxin (*n* [%])	94 (4.9%)	23 (3.6%)	21 (3.3%)	50 (7.8%)	**< 0.001**
Beta blocker (*n* [%])	1543 (79.8%)	543 (84.3%)	548 (85.1%)	452 (70.2%)	**< 0.001**
Diuretic (*n* [%])	1689 (87.4%)	595 (92.4%)	583 (90.5%)	511 (79.3%)	**< 0.001**
*Treatments*					
CRRT (*n* [%])	266 (13.8%)	71 (11.0%)	75 (11.6%)	120 (18.6%)	**< 0.001**
Invasive ventilation (*n* [%])	1386 (71.7%)	450 (69.9%)	462 (71.7%)	474 (73.6%)	0.311
PCI (*n* [%])	215 (11.1%)	68 (10.6%)	57 (8.9%)	90 (14.0%)	**0.012**
CABG (*n* [%])	62 (3.2%)	31 (4.8%)	20 (3.1%)	11 (1.7%)	**0.007**
IABP (*n* [%])	218 (11.3%)	62 (9.6%)	80 (12.4%)	76 (11.8%)	0.246
*Events*					
VT/VF (*n* [%])	329 (17.0%)	78 (12.1%)	101 (15.7%)	150 (23.3%)	**< 0.001**
Cardiac arrest (*n* [%])	233 (12.1%)	75 (11.6%)	65 (10.1%)	93 (14.4%)	0.052
Hospital mortality (*n* [%])	401 (20.7%)	72 (11.2%)	93 (14.4%)	236 (36.6%)	**< 0.001**
1‐year mortality (*n* [%])	662 (34.2%)	143 (22.2%)	166 (25.8%)	353 (54.8%)	**< 0.001**

Abbreviations: ACEI/ARB, angiotensin‐converting enzyme inhibitors/angiotensin receptor blockers; AF, atrial fibrillation; APSIII, acute physiology scores III; CABG, coronary artery bypass grafting; CKD, chronic kidney disease; COPD, chronic obstructive pulmonary disease; CRRT, continuous renal replacement therapy; FPG, fasting plasma glucose; GV, glycemic variability; HbA1c, glycosylated hemoglobin A1c; HDL, high‐density lipoprotein; HF, heart failure; IABP, intra‐aortic balloon pump; LDL, low‐density lipoprotein; LVEF, left ventricular ejection fraction; PaCO_2_, partial pressure of carbon dioxide; PaO_2_, partial pressure of oxygen; PCI, percutaneous coronary intervention; PLT, platelet count; ProBNP, *N*‐terminal pro‐brain natriuretic peptide; SAPSII, simplified acute physiology score II; SHR, stress hyperglycemia ratio; SIRS, systemic inflammatory response syndrome; SOFA, sequential organ failure assessment; TG, triglyceride; TnT, troponin T; TyG, triglyceride glucose index; VT/VF, ventricular tachycardia/ventricular fibrillation; WBC, white blood cell count.

*p* values less than 0.05 are indicated in bold.

Regarding in‐hospital treatments, patients in higher SHR tertiles more frequently received digoxin, CRRT, invasive ventilation, and PCI, but less frequently received statins, aspirin, β‐blockers, diuretics, and CABG. It is noteworthy that in the higher SHR group, the risk of VT/VF during hospitalization was higher (23.3%), but there was no difference in the risk of cardiac arrest between groups.

### 3.2 Association Between SHR, GV, TyG, and In‐Hospital Mortality

In this research, 401 (20.7%) patients died during their hospital stay. Multiple logistic regression analysis revealed that each 1‐unit increase in baseline SHR index was associated with a 51% higher risk of hospital mortality (OR: 1.51, 95% CI: 1.27–1.80, *p* < 0.001) in the unadjusted model. This association remained significant after comprehensive adjustment in the fully adjusted model (Model 2), with a 51.0% increased risk (odds ratio [OR]: 1.51, 95% CI: 1.24–1.82, *p* < 0.001). The TyG index also demonstrated stronger associations with a fully adjusted OR of 1.89 (1.50–2.39) per 1‐unit increase. In contrast, the GV index showed no significant association after full adjustment (OR: 1.00, 95% CI: 0.99–1.01, *p* = 0.473) (Table 2).

**TABLE 2:** Logistic regression showed odds ratio (OR) and 95% confidence intervals (95% CI) for association of cardiac arrest, VT/VF, hospital mortality, and 1‐year mortality in AMI patients with HFrEF.

	Unadjusted model			Adjusted Model 1			Adjusted Model 2		
	HR (95% CI)	*p* *	*p* for trend*	HR (95% CI)	*p* *	*p* for trend*	HR (95% CI)	*p* *	*p* for trend*
*Hospital mortality*									
*SHR*									
Continuous SHR index per 1 unit	1.51 (1.27–1.80)	**< 0.001**		1.39 (1.16–1.66)	**< 0.001**		1.51 (1.24–1.82)	**< 0.001**	
*T*1 (*N* = 645)	Ref.		**< 0.001**	Ref.		**< 0.001**	Ref.		**< 0.001**
*T*2 (*N* = 644)	1.34 (0.97–1.87)	0.079		0.87 (0.61–1.25)	0.447		1.16 (0.76–1.76)	0.479	
*T*3 (*N* = 644)	4.60 (3.43–6.17)	**< 0.001**		1.31 (0.93–1.84)	0.125		2.15 (1.46–1.78)	**< 0.001**	
*TyG*									
Continuous TyG index per 1 unit	1.71 (1.47–2.00)	**< 0.001**		1.30 (1.07–1.58)	**0.007**		1.89 (1.50–2.39)	**< 0.001**	
*T*1 (*N* = 645)	Ref.		**< 0.001**	Ref.		**< 0.001**	Ref.		**< 0.001**
*T*2 (*N* = 644)	1.35 (0.84–1.23)	**0.044**		0.90 (0.63–1.28)	0.552		1.59 (1.07–2.37)	**0.022**	
*T*3 (*N* = 644)	1.23 (1.02–1.48)	**< 0.001**		1.31 (0.92–1.85)	0.135		2.59 (1.72–3.91)	**< 0.001**	
*GV*									
Continuous GV index per 1 unit	1.01 (1.01–1.02)	**< 0.001**		1.01 (1.01–1.02)	**< 0.001**		1.00 (0.99–1.01)	0.473	
*T*1 (*N* = 645)	Ref.		**< 0.001**	Ref.		**< 0.001**	Ref.		**< 0.001**
*T*2 (*N* = 644)	2.08 (1.54–2.79)	**< 0.001**		2.07 (0.99–1.46)	**< 0.001**		1.81 (1.20–2.71)	**0.004**	
*T*3 (*N* = 644)	2.54 (1.90–3.39)	**< 0.001**		2.80 (2.03–3.86)	**< 0.001**		1.62 (1.06–2.47)	**0.025**	
*Cardiac arrest*									
*SHR*									
Continuous SHR index per 1 unit	1.00 (0.97–1.03)	0.985		1.00 (0.97–1.03)	0.991		0.93 (0.77–1.13)	0.482	
*T*1 (*N* = 645)	Ref.		0.054	Ref.		**< 0.001**	Ref.		**< 0.001**
*T*2 (*N* = 644)	0.85 (0.98–2.10)	0.376		1.51 (1.03–2.22)	**0.034**		0.85 (0.58–1.24)	0.472	
*T3* (*N* = 644)	1.28 (0.93–1.78)	0.134		1.52 (0.99–2.31)	0.052		0.87 (0.60–1.27)	0.403	
*TyG*									
Continuous TyG index per 1 unit	1.30 (1.09–1.55)	**0.004**		1.09 (1.01–1.19)	**0.036**		1.21 (0.98–1.49)	0.076	
*T*1 (*N* = 645)	Ref.		0.069	Ref.		**< 0.001**	Ref.		**< 0.001**
*T*2 (*N* = 644)	0.91 (0.64–1.29)	0.598		0.80 (0.56–1.16)	0.241		0.88 (0.60–1.29)	0.504	
*T*3 (*N* = 644)	1.32 (0.95–1.84)	0.095		0.85 (0.59–1.23)	0.401		1.16 (0.79–1.70)	0.458	
*GV*									
Continuous GV index per 1 unit	1.00 (0.99–1.01)	0.087		1.01 (0.99–1.01)	0.117		1.00 (0.99–1.01)	0.777	
*T*1 (*N* = 645)	Ref.		**0.011**	Ref.		**< 0.001**	Ref.		**< 0.001**
*T*2 (*N* = 644)	1.64 (1.08–2.19)	**0.005**		1.70 (1.19–2.45)	**0.004**		1.49 (1.01–2.21)	**0.046**	
*T*3 (*N* = 644)	1.53 (1.08–2.19)	**0.017**		1.56 (1.06–2.30)	**0.024**		1.10 (0.72–1.68)	0.675	
*VT/VF*									
*SHR*									
Continuous SHR index per 1 unit	1.50 (1.29–1.74)	**< 0.001**		1.46 (1.25–1.71)	**< 0.001**		1.28 (1.08–1.52)	**0.005**	
*T*1 (*N* = 645)	Ref.		**< 0.001**	Ref.		**< 0.001**	Ref.		**< 0.001**
*T*2 (*N* = 644)	1.35 (0.98–1.85)	0.063		1.35 (0.97–1.87)	0.072		1.38 (1.08–1.95)	**0.037**	
*T*3 (*N* = 644)	2.21 (1.64–2.98)	**< 0.001**		2.11 (1.54–2.88)	**< 0.001**		1.65 (1.16–2.33)	**0.005**	
*TyG*									
Continuous TyG index per 1 unit	1.25 (1.07–1.45)	**0.004**		1.35 (1.14–1.60)	**< 0.001**		1.35 (1.12–1.64)	**0.002**	
*T*1 (*N* = 645)	Ref.		**0.034**	Ref.		**< 0.001**	Ref.		**< 0.001**
*T2* (*N* = 644)	0.97 (0.71–1.31)	0.826		1.01 (0.74–1.38)	0.940		1.04 (0.74–1.46)	0.807	
*T*3 (*N* = 644)	1.36 (1.02–1.81)	**0.034**		1.55 (1.14–2.11)	**0.005**		1.50 (1.06–2.12)	**0.023**	
*GV*									
Continuous GV index per 1 unit	1.32 (1.00–1.01)	**0.021**		1.01 (1.00–1.02)	**0.018**		1.01 (0.99–1.01)	0.107	
*T*1 (*N* = 645)	Ref.		**0.002**	Ref.		**< 0.001**	Ref.		**< 0.001**
*T*2 (*N* = 644)	1.32 (0.97–1.80)	0.073		1.25 (0.91–1.72)	0.174		1.09 (0.77–1.54)	0.621	
*T*3 (*N* = 644)	1.71 (1.27–2.30)	**< 0.001**		1.76 (1.26–1.45)	**0.001**		1.37 (0.95–1.99)	0.091	
*1‐year mortality*									
*SHR*									
Continuous SHR index per 1 unit	2.94 (2.25–3.82)	**< 0.001**		2.78 (2.11–3.66)	**< 0.001**		1.40 (1.19–1.65)	**< 0.001**	
*T*1 (*N* = 645)	Ref.		**< 0.001**	Ref.		**< 0.001**	Ref.		< 0.001
*T*2 (*N* = 644)	1.22 (0.94–1.56)	0.130		1.22 (0.93–1.61)	0.147		1.13 (0.80–1.59)	0.490	
*T*3 (*N* = 644)	4.26 (3.34–5.42)	**< 0.001**		4.01 (3.09–5.21)	**< 0.001**		1.92 (1.39–2.67)	**< 0.001**	
*TyG*									
Continuous TyG index per 1 unit	1.47 (1.30–1.67)	**< 0.001**		1.82 (1.57–2.11)	**< 0.001**		1.33 (1.10–1.60)	**0.003**	
*T*1 (*N* = 645)	Ref.		**< 0.001**	Ref.		**< 0.001**	Ref.		**< 0.001**
*T*2 (*N* = 644)	1.33 (1.06–1.69)	**0.016**		1.48 (1.15–1.91)	**0.002**		1.26 (0.91–1.75)	0.163	
*T*3 (*N* = 644)	1.70 (1.35–2.14)	**< 0.001**		2.28 (1.75–2.97)	**< 0.001**		1.27 (0.86–1.81)	0.195	
*GV*									
Continuous GV index per 1 unit	1.03 (1.02–1.04)	**< 0.001**		1.02 (1.02–1.04)	< **0.001**		1.02 (1.01–1.02)	**< 0.001**	
*T*1 (*N* = 645)	Ref.		**< 0.001**	Ref.		**< 0.001**	Ref.		**< 0.001**
*T*2 (*N* = 644)	2.42 (1.89–3.11)	**< 0.001**		2.29 (1.75–3.00)	**< 0.001**		1.90 (0.68–1.53)	**< 0.001**	
*T*3 (*N* = 644)	3.27 (2.56–4.20)	**< 0.001**		3.34 (2.52–4.43)	**< 0.001**		1.89 (1.32–2.72)	**0.001**	

*Note:* Model 1: Adjusted for age, gender, race, diabetes, stroke, hypertension, HF, AF, CKD, and COPD.

* *p* values and *p* for trend less than 0.05 are indicated in bold.

Model 2: Adjusted for Model 1 + BMI, SBP, hemoglobin, WBC, PLT, creatinine, PaO_2_, LDL, HDL, TnT, ProBNP, LVEF, SOFA, SIRS, SAPSII, APSIII, ACEI/ARB, beta‐blockers, digoxin, statin, aspirin, diuretic, CRRT, invasive ventilation, PCI, CABG, and IABP.

When analyzed by tertiles, patients in the highest SHR quartile (*T*3) exhibited significantly increased in‐hospital mortality compared to those in the lowest tertile (*T*1), with an adjusted OR of 2.15 (1.46–1.78, *p* < 0.001). This pattern was more pronounced for TyG, with an adjusted OR of 2.59 (1.72–3.91, *p* < 0.001) for *T*3 versus *T*1. It is worth noting that GV tertiles showed statistically significant association with in‐hospital mortality after full adjustment for *T*3 (OR: 1.62, 95% CI: 1.06–2.47, *p* = 0.025) and *T*2 (OR: 1.81, 95% CI: 1.20–2.71, *p* = 0.025) versus *T*1 (Table 2, Figure S2A).

**FIGURE 1:**
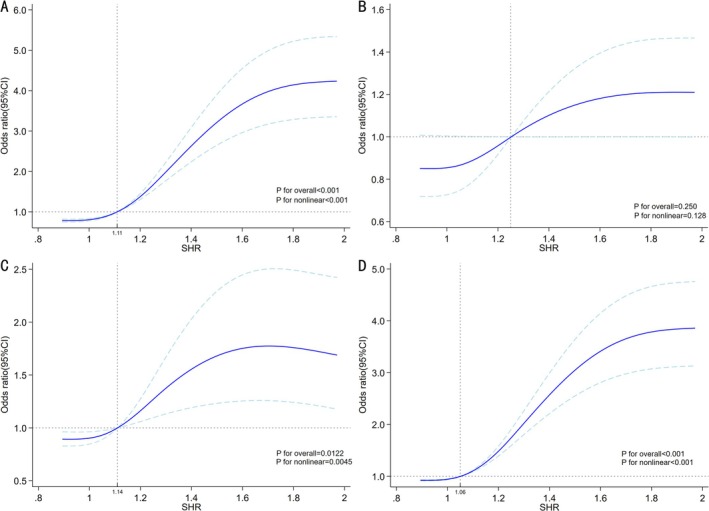
RCS model analysis of the associations of the SHR with different endpoints in the total study population. (A) In‐hospital mortality. (B) Cardiac arrest. (C) Ventricular tachycardia/ventricular fibrillation. (D) One‐year mortality.

RCS model analysis confirmed the positive dose–response associations of SHR indices (*p* for overall < 0.001, *p* for nonlinear < 0.001), indicating that elevated values of indices corresponded to progressively increased in‐hospital mortality risk (Figure 1A).

### 3.3 Association Between SHR, GV, TyG, and 1‐Year Mortality

Among hospitalized patients with AMI and LVEF < 40%, 662 patients (34.2%) died within 1 year of follow‐up. When analyzed as a continuous variable, each 1‐unit increase in the SHR index was associated with a 2.94‐fold higher risk of 1‐year mortality (OR: 2.94, 95% CI: 2.25–3.82, *p* < 0.001) in the unadjusted model. This association remained significant after comprehensive adjustment for potential confounders (Model 1: OR = 2.78 [2.11–3.66], *p* < 0.001; Model 2: OR = 1.40 [1.19–1.65], *p* < 0.001). The TyG and GV indices also showed significant associations with mortality in the unadjusted model (OR = 1.47, 95% CI: 1.30–1.67, *p* < 0.001 and OR = 1.03, 95% CI: 1.02–1.04, *p* < 0.001), and maintained significant associations after full adjustment (TyG: OR = 1.33, 95% CI: 1.10–1.60, *p* = 0.003; GV: OR = 1.02, 95% CI: 1.01–1.02, *p* < 0.001) (Table 2).

When the three indices were categorized by tertiles, patients in the highest SHR and GV tertiles (*T*3) exhibited significantly increased 1‐year mortality risk compared to those in the lowest tertile (*T*1), with this association persisting after full adjustment (SHR: OR = 1.92 [1.39–2.67], *p* < 0.001; GV: OR = 1.89 [1.32–2.72], *p* = 0.001). Notably, TyG demonstrated no significant associations with 1‐year mortality when analyzed by tertiles, with no evidence of a progressive risk increase across higher tertiles for TyG after full adjustment (OR: 0.195, 95% CI: 0.86–1.81, *p* = 0.195) (Table 2, Figure S2B).

RCS model analysis demonstrated that the SHR index also showed a positive correlation with 1‐year mortality (*p* for overall < 0.001, *p* for nonlinear < 0.001), indicating that elevated values of indices corresponded to progressively increased 1‐year mortality risk (Figure 1D).

### 3.4 Predictive Value of Three Indices for Cardiac Arrest and VT/VF

**FIGURE 2:**
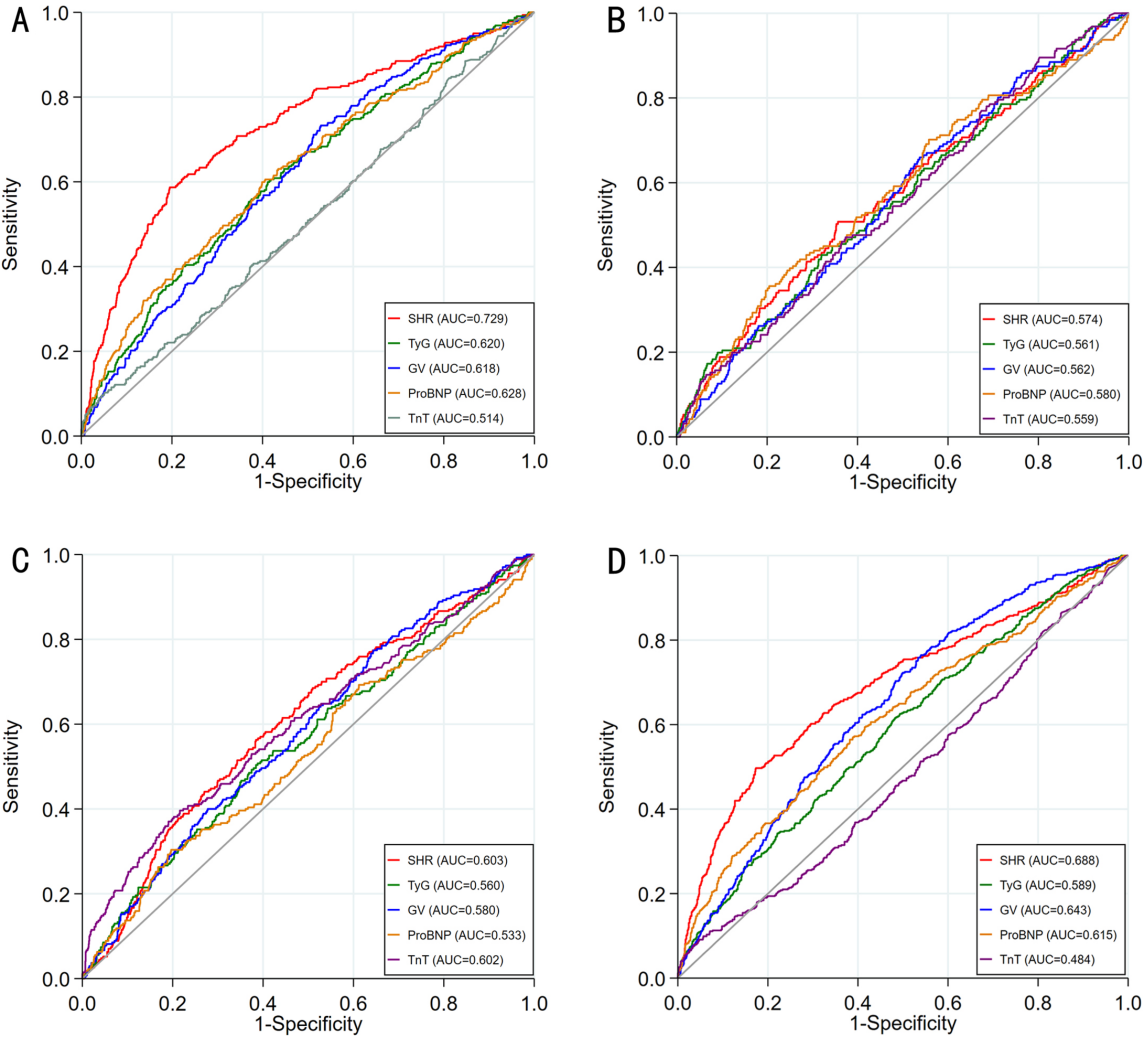
ROC curves of SHR, TyG and GV as markers for predicting the risk of different endpoints. (A) In‐hospital mortality, DeLong test *p* < 0.001. (B) Cardiac arrest, DeLong test *p* = 0825. (C) ventricular tachycardia/ventricular fibrillation, DeLong test *p* = 0.033. (D) One‐year mortality, DeLong test *p* < 0.001.

Figure 2 illustrates the ROC curves comparing the predictive performance of SHR, TyG, and GV indices for incidence compared to traditional clinical markers like TnT and ProBNP. For predicting in‐hospital mortality, SHR (AUC: 0.729, 95% CI: 0.689–0.751) demonstrated better discriminative ability than TyG (AUC: 0.620, 95% CI: 0.580–0.642), GV (AUC: 0.618, 95% CI: 0.588–0.652), ProBNP (AUC: 0.628, 95% CI: 0.593–0.656), and TnT (AUC: 0.514, 95% CI: 0.479–0.544) (DeLong test *p* < 0.001) (Figure 2A). When predicting 1‐year all‐cause mortality in these low LVEF AMI patients, the SHR index (AUC: 0.688, 95% CI: 0.661–0.713) demonstrated significantly superior discriminative ability compared to the other indices (TyG 0.589 [0.562–0.615]; GV 0.643 [0.619–0.660]; ProBNP 0.615 [0.590–0.644]; TnT 0.484 [0.451–0.531]; DeLong test *p* < 0.001) (Figure 2D).

We further evaluated the predictive ability of these indexes to cardiac arrest and VT/VF during hospitalization in patients with low EF after AMI. In cardiac arrest, SHR (AUC: 0.574, 95% CI: 0.527–0.610), TyG (AUC: 0.561, 95% CI: 0.526–0.606) and GV (AUC: 0.562, 95% CI: 0.520–0.610) indices do not perform better predictive ability (DeLong test *p* = 0.825), while compared to ProBNP and TnT (Figure 2B). However, for VT/VF prediction, the predictive power of SHR (AUC: 0.603, 95% CI: 0.561–0.630) was superior to TyG (AUC: 0.560, 95% CI: 0.511–0.581) and GV (AUC: 0.565, 95% CI: 0.532–0.598), but similar to that of TnT (AUC: 0.602, 95% CI: 0.560–0.631), with no statistically significant differences between groups (DeLong test *p* = 0.033). (Figure 2C).

When we further employed the RCS model to analyze the dose–response relationship between SHR index and cardiac arrest and VT/VF events, we observed a positive correlation between SHR and VT/VF events (*p* for overall < 0.012, *p* for nonlinear < 0.0045). However, no significant dose–response trend was identified between SHR and in‐hospital cardiac arrest events (*p* for overall < 0.250, *p* for nonlinear < 0.128), indicating that elevated SHR index values correspond to a progressive increase in the risk of in‐hospital VT/VF occurrence (Figure 1B,C).

### 3.5 Subgroup Analyses of SHR and Outcomes

**FIGURE 3:**
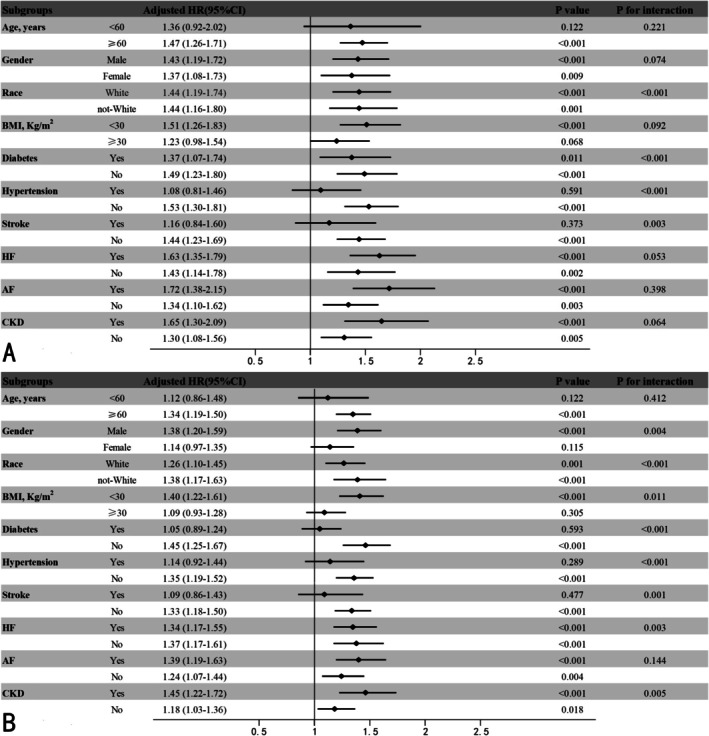
Subgroup analysis of the association between the SHR index and mortality. (A) Analysis of the association between the SHR index and the risk of in‐hospital mortality. (B) Analysis of the association between the SHR index and one‐year mortality in AMI patients with low LVEF.

To assess the consistency of associations between SHR and mortality, we conducted subgroup analyses for two endpoints, with results presented in Figure 3. In the study of the primary endpoint, the association between baseline SHR and hospital mortality remained consistent across subgroups stratified by age (< 60 or ≥ 60 years), gender, BMI (< 30 or ≥ 30 kg/m^2^), heart failure, atrial fibrillation, and CKD status (*p* for interaction > 0.05). However, significant interactions were observed with race (*p* for interaction < 0.001), diabetes (*p* for interaction < 0.001), hypertension (*p* for interaction < 0.001), and stroke (*p* for interaction = 0.003) in relation to hospital mortality (Figure 3A).

When analyzed, 1‐year mortality among these patients (Figure 3B), when participants were stratified by gender and atrial fibrillation, the association between mean SHR index and 1‐year mortality remained consistent (*p* for interaction > 0.05). Significant interactions were observed within gender (*p* for interaction = 0.004), race (*p* for interaction < 0.001), BMI (*p* for interaction = 0.011), diabetes (*p* for interaction < 0.001), hypertension (*p* for interaction < 0.001), stroke (*p* for interaction = 0.001), heart failure (*p* for interaction = 0.003), and CKD subgroups (*p* for interaction = 0.005).

## 4 Discussion

This study provides important evidence supporting the integration of readily available metabolic markers into individualized risk assessment strategies for patients with reduced ejection fraction following AMI.

### 4.1 SHR and Prognosis After Myocardial Infarction

Stress hyperglycemia is a common metabolic disorder in critically ill patients, reflecting the body's response to acute stress. Previous studies have shown that hyperglycemia at admission is associated with poor prognosis in various acute diseases, but traditional glucose measurements fail to adequately consider the patient's baseline glycemic status [18, 19, 20]. The SHR, as an indicator for assessing stress‐related hyperglycemia, more accurately reflects the intensity of the stress response by calculating the ratio of acute glucose levels to estimated average glucose levels [21].

Interestingly, some previous studies have found that stress hyperglycemia in AMI patients is more likely to be associated with worse prognosis compared to patients with known diabetes [22]. A recent observational study of 6287 STEMI patients demonstrated that the highest SHR quartile was significantly associated with adverse outcomes in non‐diabetic patients (but not in diabetic patients) during a 5‐year follow‐up period [23]. Our study is the first to systematically compare the predictive value of SHR, GV, and TyG in patients with reduced ejection fraction after AMI, finding that SHR performs best in predicting in‐hospital and 1‐year all‐cause mortality.

### 4.2 Limitations of Previous SHR Studies

Although SHR has shown prognostic value in various cardiovascular diseases, previous studies have several limitations. Yang et al. demonstrated that SHR has a U‐shaped association with major cardiovascular and cerebrovascular events in patients with acute coronary syndrome after drug‐eluting stent implantation [24]. Wei et al. also observed a J‐shaped relationship between stress hyperglycemia and mortality outcomes in ACS patients undergoing PCI [25]. Gao et al. found that SHR was closely related to in‐hospital major adverse cardiovascular events in ST‐segment elevation myocardial infarction patients [21].

However, these studies often used different SHR thresholds and grouping methods, lacking standardized risk stratification criteria; rarely simultaneously evaluated the relative predictive value of SHR and other glycemic indices [26]; mainly focused on overall mortality or composite endpoints, with insufficient exploration of specific cardiovascular events such as arrhythmias [27]; concentrated on general cardiovascular patient populations, with limited research on specific high‐risk subgroups such as AMI patients with reduced ejection fraction [28]; and primarily assessed short‐term outcomes, lacking systematic evaluation of SHR's ability to predict long‐term prognosis [29]. Our study addresses these research gaps by simultaneously evaluating the predictive value of three metabolic indices for short‐term and long‐term mortality and arrhythmic events.

### 4.3 Mechanisms Linking SHR With Poor Prognosis After Myocardial Infarction

Multiple mechanisms may explain the association between SHR and poor prognosis after AMI. Stress hyperglycemia exacerbates myocardial injury and promotes adverse remodeling by promoting inflammatory responses, aggravating endothelial dysfunction, enhancing platelet aggregation, and reducing fibrinolytic activity [30, 31]. Mild to moderate stress hyperglycemia may play a protective role in the acute phase by increasing cell survival factors and decreasing apoptosis [32, 33], but excessive SHR may trigger inflammation and oxidative stress, aggravate endothelial dysfunction, and induce a prothrombotic state [11, 13, 30].

During myocardial infarction, stress hyperglycemia promotes a prothrombotic state and activates the neuroendocrine system, causing excessive release of catecholamines and cytokines, damaging vascular endothelial function [34, 35]. Additionally, stress hyperglycemia may lead to increased production of reactive oxygen species, inducing cardiomyocyte apoptosis and cardiac dysfunction [36]. Even transient episodes of stress hyperglycemia can affect long‐term cardiovascular outcomes through epigenetic modifications—a phenomenon known as “metabolic memory,” which may explain why SHR has significant predictive value for both short‐term and long‐term mortality, especially in non‐diabetic patients [34, 37].

### 4.4 Unique Value of This Study in Patients With Reduced LVEF

This study is the first to systematically evaluate the prognostic predictive value of SHR in AMI patients with LVEF below 40%, a specific population representing the highest‐risk subgroup after myocardial infarction. AMI patients with reduced ejection fraction face significantly increased risk of sudden cardiac death, mainly due to ventricular remodeling, increased electrical instability, and neurohumoral activation. In this high‐risk population, accurate risk stratification is essential for guiding preventive interventions, such as implantable cardioverter‐defibrillators or wearable cardioverter‐defibrillators.

Our study shows that in AMI patients with reduced LVEF, SHR is not only a strong predictor of all‐cause mortality but also significantly associated with the risk of VT/VF. This finding has important clinical implications because VT/VF is the main mechanism of sudden death in these patients. Unlike previous studies, our analysis specifically focused on the association between SHR and fatal arrhythmias, providing an additional predictive dimension in assessing the risk of sudden death. Furthermore, we found that the predictive value of SHR is more significant in non‐diabetic patients, consistent with previous research findings, suggesting that stress hyperglycemia may have different pathophysiological significance in patients with different baseline metabolic states [23].

Notably, our study simultaneously evaluated three metabolic indices (SHR, GV, and TyG) and found that SHR performs best in predicting mortality. This result supports the prioritized use of SHR for risk assessment in AMI patients with reduced LVEF. Compared to relying solely on admission glucose or HbA1c, SHR provides a more comprehensive assessment of stress response by considering the degree of acute glucose elevation relative to baseline glycemic status, particularly suitable for these high‐risk patients with significant myocardial damage and neuroendocrine activation.

### 4.5 Clinical Application Prospects

These findings have important clinical implications. The superior predictive performance of SHR suggests that this index should be incorporated into risk stratification models for patients with reduced ejection fraction after AMI. Regular monitoring of SHR during ICU stay can identify high‐risk individuals who may benefit from more intensive monitoring and individualized treatment. Based on our results, optimal glycemic management should aim to minimize stress hyperglycemia while avoiding hypoglycemia and excessive glucose fluctuations.

Previous research has confirmed that stress hyperglycemia is prevalent among AMI patients, even in non‐diabetic individuals, and targeted preventive strategies have demonstrated benefits in these patients [38, 39]. In this study for AMI patients with LVEF below 40%, we recommend SHR as a routine assessment index, especially in evaluating the risk of sudden death and guiding preventive interventions. Future research should explore whether interventions targeting stress hyperglycemia can improve the prognosis of this high‐risk population and determine the optimal SHR thresholds to guide clinical decision‐making. Additionally, prospective studies need to evaluate whether SHR‐guided management strategies can reduce mortality and arrhythmic complications in AMI patients with reduced LVEF.

## 5 Conclusions and Limitations

This study represents the first comprehensive comparison of three glucose metabolism disorder indices (SHR, GV, and TyG) for predicting mortality and arrhythmic risk in patients with reduced ejection fraction following AMI. Our findings demonstrate that SHR exhibits superior performance in predicting both in‐hospital and 1‐year all‐cause mortality, significantly outperforming GV and TyG indices. Additionally, SHR demonstrated better discriminative ability for VT/VF risk, while none of the three indices showed significant predictive value for cardiac arrest. Utilizing SHR to identify stress hyperglycemia may facilitate early identification of high‐risk individuals and implementation of targeted interventions, potentially reducing mortality risk and arrhythmic events in post‐AMI patients.

Despite these strengths, several limitations warrant consideration. First, as a retrospective study, although we adjusted for numerous potential confounders using multivariate logistic regression and Cox proportional hazards regression models, residual confounding factors may persist and cannot be completely eliminated. Second, this study was conducted in a single database without external validation, and the generalizability of our findings requires further verification across different populations and healthcare settings. Finally, while we explored the dose–response relationship between SHR and outcomes, we were unable to determine optimal threshold values for clinical decision‐making, which need further exploration in future studies.

## Author Contributions

Weiyan Lai and Xiuying Peng analyzed and interpreted patient data and were major contributors to writing the manuscript. Hui Peng collected the data and participated in editing the manuscript. Yang Chen contributed to the conception and design of this study and editing of the manuscript. All authors read and approved the final manuscript.

## Ethics Statement

Because the MIMIC database is publicly available, no additional ethical approval or informed consent was required for this study. The researchers completed an online course from the National Institutes of Health and successfully passed the relevant exam (application number 60069524) before using the MIMIC database. One of the study participants was given access to the ARIC database before this study began. Because all ARIC study participants provided written informed consent before the study interview and the database was anonymous, no additional ethical approval or informed consent was required for this specific study.

## Consent

The authors have nothing to report.

## Conflicts of Interest

The authors declare no conflicts of interest.

## Supporting information


**Figure S1.** Selection flowchart for study participants. AMI, acute myocardial infarction; GV, glycemic variability; ICD, International Classification of Diseases; ICU, intensive care unit; LVEF, left ventricular ejection fraction; MIMIC‐IV, Medical Information Mart for Intensive Care IV; SHR, stress hyperglycemia ratio; TyG, triglyceride‐glucose index.

**Figure S2.** Kaplan–Meier survival analysis curves by tertiles of SHR, illustrating the mortality incidence. (A) In‐hospital mortality, log‐rank test *p* < 0.001. (B) One‐year mortality, log‐rank test *p* < 0.001.

## Data Availability

The data utilized in this study were derived from the MIMIC‐IV database (version 3.1). The analyzed datasets are available from the corresponding author upon reasonable request, subject to appropriate data use agreements and ethical approvals.
