# Supplementary figures and images for: Stress Hyperglycemia Ratio Outperforms Glycemic Variability in Predicting Mortality Among Acute Myocardial Infarction Patients With Reduced Ejection Fraction: A Retrospective Cohort Study
Source: J Diabetes. 2025 Aug 13;17(8):e70122. doi: 10.1111/1753-0407.70122 (PMC12350036; doi:10.1111/1753-0407.70122)

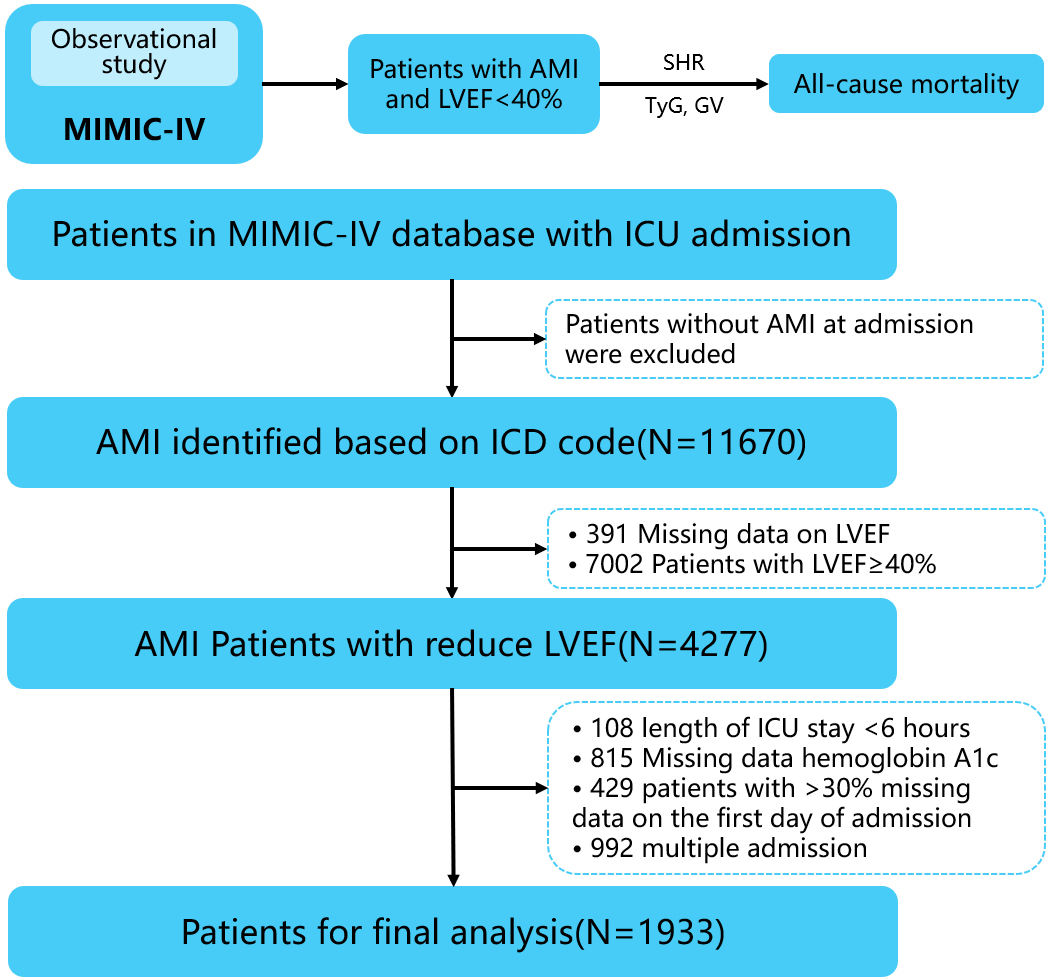

Supplement: Supplementary file 1 — Figure S1. Selection flowchart for study participants. AMI, acute myocardial infarction; GV, glycemic variability; ICD, International Classification of Diseases; ICU, intensive care unit; LVEF, left ventricular ejection fraction; MIMIC‐IV, Medical Information Mart for Intensive Care IV; SHR, stress hyperglycemia ratio; TyG, triglyceride‐glucose index. [file JDB-17-e70122-s002.jpg]

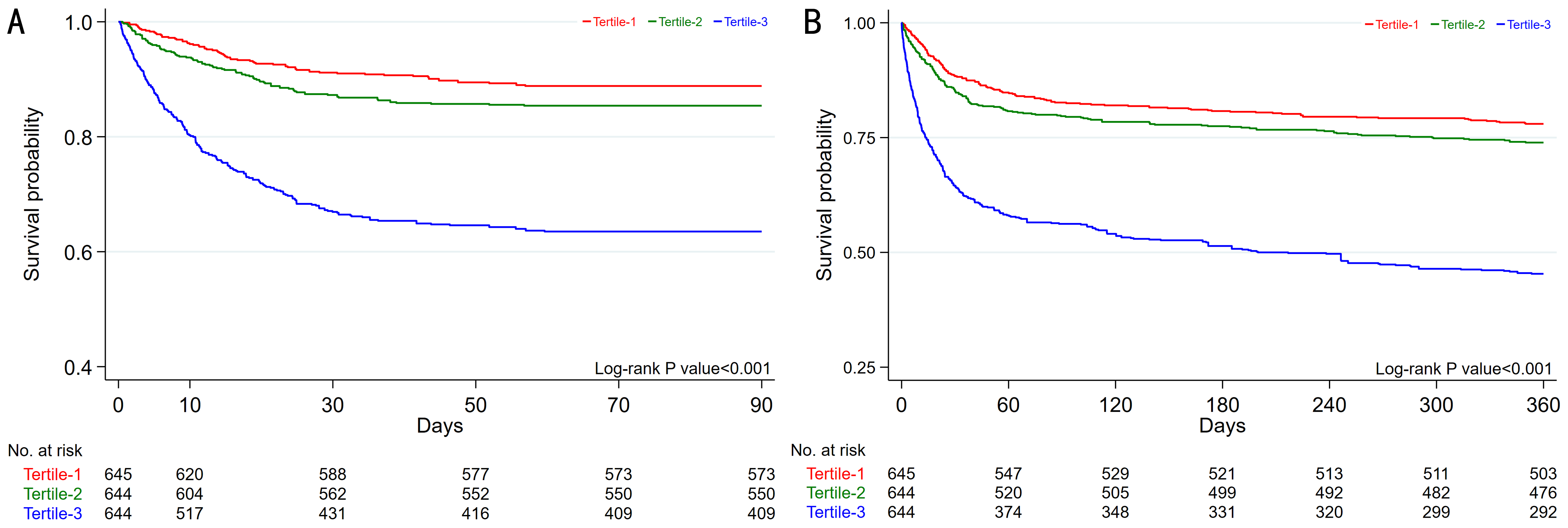

Supplement: Supplementary file 2 — Figure S2. Kaplan–Meier survival analysis curves by tertiles of SHR, illustrating the mortality incidence. (A) In‐hospital mortality, log‐rank test p < 0.001. (B) One‐year mortality, log‐rank test p < 0.001. [file JDB-17-e70122-s001.jpg]
